# Dry Gangrene

**Published:** 1856-09

**Authors:** Charles William Ashby

**Affiliations:** Alexandria, Virginia


ATLANTA
Medical and Surgical Journal.

Vol. II.] SEPTEMBER, 1856. [No. 1.

### ORIGINAL COMMUNICATIONS.

### ARTICLE I.

*Dry Gangrene.* By Charles William Ashby, M. D., Alexandria, Virginia.

This is quite a rare disease, and therefore, it may be that a report of a case which has recently occurred in my practice, may not be without some interest to the profession.

Sometime in April I was called to see Mrs. Hall, and found her suffering, occasionally, intense pain in one toe, and at times, also, in the foot. I was so forcibly struck with the entire insensibility of the foot to the touch, its intense cadaveric coldness and diffused bluish-redness or ecchymotic appearance, that I felt sure it was a serious case of disease, and expressed the opinion that it would probably result in Gangrene, such as is so accurately described by Pott and others. It had been suggested that it was a case of frost-bite, but the old lady protested she had not been exposed in the slightest degree. She had been suffering about four weeks when I first saw her; she was taken with pain in the night, and discovered the coldness, insensibility and redness the next morning; she was 67 years old. I put the old lady on a good generous diet, with the free use of opium and mild local applications. She continued this treatment for several weeks; and although the pain was greatly relieved by the opium, there was no improvement in the appearance or character of the disease.

As I could not conscientiously hold out much hope as to her recovery, she, for a time, went into the hands of a quack, who decided, very positively, that it was a frost-bite, and promised a speedy cure.

As I was unwilling to lose sight of the case, I requested Jno. Danforth, a student of mine, to call and see her occasionally, and watch the progress of the disease. It gradually increased, probably aggravated by the harsh treatment, and in about two weeks a small black spot appeared on the second toe, which confirmed the original diagnosis.

She now again became my patient, and from this time (now about two months from the first appearance of the disease) I witnessed, with painful anxiety, its rapid progress: first one toe becoming black and dry, and then another, until nearly the entire foot was implicated.

I had attended this old lady some six months previously for a hemorrhage of the lungs, but her general health now seemed to be very good; she had no cough, nor indeed any constitutional symptom up to this time; she was very poor, and had undergone a great deal of trouble and hardship in life.

Notwithstanding the opinions of the older surgeons to the contrary, Sir Astley Cooper among the number, after a free and full consultation of the entire faculty of Alexandria, it was unanimously agreed that amputation above the knee held out the only possible hope of recovery. This decision was based upon the more recent report of some successful cases, where amputation, *high up the thigh*, was practised.

There was little or no discharge or fœtid odor, and the mummy-like appearance was very striking. There was no well marked line of separation, but on the day of the operation, for the first time, I observed red lines passing up the leg: this I regarded as most unfavorable.

After a fair representation of the almost hopelessness of her case, she was anxious for the operation. It was done in the usual way, in the presence of Drs. Fairfax, Murphy, Lewis, French, Brown, Chancellor and Gregory.

Under the influence of chloroform she suffered not at all; and there was no apparent shock to the system, the pulse being only 80 in the minute, after she was put to bed; very little blood was lost, there being no jet from the femoral artery.

The tenaculum in the mouth of the cut femoral artery at once arrested my attention to the grating sound of ossific deposit.

At the first dressing, on the third day, unmistakable indications were present that the disease had attacked the stump.

Although she was apparently quite comfortable, enjoyed her food, and was very cheerful up to the 10th day, she had a red glazed and dry tongue nearly the whole time. At this period, she was taken with intense pain in the other foot, and the deadness, coldness and peculiar redness which soon appeared, satisfied her that it was the same disease in that limb also. She now gave up all hope of recovery, and sank rapidly on 13th day after the operation.

The examination of the artery, after the operation, exhibited *its entire closure* by a semi-calcarious deposit, commencing at the popliteal region. At the post mortem, conducted by my intelligent young friend, Dr. Wm. Gregory, the femoral artery was found very much contracted about two inches above the operation, had various patches of ossific deposit, and a recent clot up to the profunda artery. The profunda was inordinately enlarged, doubtless from its having conducted for some time past, the almost entire circulation.

This brief and imperfect report is given merely that the profession may judge as to the propriety of operating in such cases.

The publication of our unfortunate cases, and even our admitted errors in practice, may serve as beacons for others, and thus prove most valuable.

After a review of all the circumstances, I do not hesitate to say that I would perform the same operation again, as I believe that amputation *high up the thigh* affords the only rational hope of recovery.

